# Phytochemical composition, anti-inflammatory activity and cytotoxic effects of essential oils from three *Pinus* spp.

**DOI:** 10.1080/13880209.2017.1309555

**Published:** 2017-04-07

**Authors:** Mimoza Basholli-Salihu, Roswitha Schuster, Avni Hajdari, Dafina Mulla, Helmut Viernstein, Behxhet Mustafa, Monika Mueller

**Affiliations:** a Department of Pharmaceutical Technology and Biopharmaceutics, University of Vienna, Vienna, Austria;; b Department of Pharmacy, Faculty of Medicine, University of Prishtina, Prishtina, Kosovo;; c Department of Biology, Faculty of Mathematical and Natural Science, University of Prishtina, Pristhina, Kosovo;; d Institute of Biological and Environmental Research, University of Prishtina, Prishtinë, Kosovo

**Keywords:** *Pinus mugo*, *Pinus peuce*, *Pinus heldreichii*, inflammation, cancer

## Abstract

**Context:** Inflammation and cell differentiation lead to a number of severe diseases. In the recent years, various studies focused on the anti-inflammatory and anticancer activity of essential oils (EOs) of numerous plants, including different *Pinus* species.

**Objective:** The phytochemical composition, anti-inflammatory and cytotoxic activity of EOs from needles and twigs of *Pinus heldreichii* Christ (Pinaceae) and *P. peuce* Griseb., and from needles, twigs and cones of *P. mugo* Turra were determined.

**Materials and methods:** For separation and identification of the EOs, gas chromatography/flame ion detector (GC/FID) and GC/mass spectrometry were performed. The amount of secreted IL-6 in a lipopolysaccharide (LPS)-stimulated macrophage model was quantified (concentration of oils: 0.0001–0.2%, 3 h incubation). Cytotoxicity on the cancer cell lines HeLa, CaCo-2 and MCF-7 were determined using a MTT (Thiazolyl Blue Tetrazolium Bromide) assay (concentration of oils: 0.001–0.1%, 24 h incubation).

**Results:** The most prominent members in the oils include: δ-3-carene, α-pinene and linalool-acetate (*P. mugo*); α-pinene, β-phellandrene and β-pinene (*P. peuce*); limonene, α-pinene and (E)-caryophyllene (*P. heldreichii*). EOs showed significant cytotoxic effects on cancer cell lines (IC_50_ 0.007 to >0.1%), with a reduction in cell viability with up to 90% at a concentration of 0.1%, and anti-inflammatory activity (IC_50_ 0.0008–0.02%) with a reduction of IL-6 secretion with up to 60% at a concentration of 0.01%.

**Discussion and conclusion:** The EOs of needles and twigs from *P. peuce* and *P. heldreichii* as well as of needles, twigs and cones of *P. mugo* can be considered as promising agents for anticancer and anti-inflammatory drugs.

## Introduction

Essential oils (EOs) are plant volatile oils that have been widely used in traditional medicine. Recently, EOs biological activity (antimicrobial, anti-inflammatory, antifungal, antiviral, antioxidant and anticancer) was shown in scientific studies (Bakkali et al. 2008; Adorjan & Buchbauer 2010; Bayala et al. 2014). The chemical composition of the EOs is affected by many factors, such as genetic variation, geographic location, climate, season, stress during growth, maturity, drying and storage, thus influencing their biological properties (Croteau 1986; Hussain et al. 2008; Raut & Karuppayil 2014).

Inflammatory and oxidative disorders lead to a number of diseases including cancer (Lahlou 2004; Bayala et al. 2014). Cancer belongs to the second largest diseases, causing more than 10% of deaths worldwide (Loizzo et al. 2008). Several drugs are available as anti-inflammatory (i.e., non-steroidal anti-inflammatory drugs (NSAIDs) and corticosteroids) and anticancer agents. Treatment of cancer with chemotherapy and/or radiation is associated with high systemic toxicity and development of drug resistance, resulting in unsuccessful treatment (Buchholz & Gress 2009). Medical treatment of inflammatory disorders provides an effective curative response, but it is frequently connected to several side effects due to its regular use (Perrone et al. 2003; Hippisley-Cox & Coupland 2005). This led to an increased interest of research in considering plant extracts as potential source for the development of novel alternative anticancer or anti-inflammatory agents (Efferth et al. 2007).

Recent studies reported that EOs from some plants reduce and inhibit the production of inflammatory mediators by different mechanisms and exert anti-proliferative and cytotoxic effects against cancer cells (Adorjan & Buchbauer 2010). For example, the EOs of *Abies koreana* E.H.Wilson (Pinaceae) showed a significant inhibition of the pro-inflammatory mediators IL-1β, IL-6, TNF-α, NO and PGE_2_ in LPS-stimulated macrophages (Yoon et al. 2009). *Illicium anisatum* L. (Illiciaceae) EO decreased the expression of iNOS and COX-2 and is additionally an effective inhibitor of LPS-induced PGE_2_ and NO production in macrophages (Kim et al. 2009). The EOs of *Cymbopogon flexuosus* Stapf. (Poaceae) showed anticancer activity against 502713 (colon) and IMR-32 (neuroblastoma) cell lines and led to apoptosis in HL-60 (promyeloblast) cell lines (Sharma et al. 2009). *Anemopsis californica* (Nutt.) Hook. & Arn. (Saururaceae) EOs showed anti-proliferative activity of AN3CA (uterine) and HeLa (cervical) cell lines; EOs such as β-caryophyllene and α-humulene exerted significant anticancer activity in MCF-7 (breast) and DLD-1 (colon) cell lines (Legault & Pichette 2007; Medina-Holguín et al. 2008).

There are several studies investigating the chemical composition of the EOs of selected parts of *Pinus mugo* Turra (Pinaceae), *P. peuce* Griseb. and *P. heldreichii* Christ obtained from Kosovo flora, but to the best of our knowledge there are, as yet, no reports about the anti-inflammatory activity and cytotoxic effects of the EOs from these *Pinus spp.* (Stevanović-Janežić & Vilotić 1998; Hajdari et al. 2015). In this study, we assess the anti-inflammatory and cytotoxic potential of the EOs of *P. mugo* (needles, twigs and cones), *P. peuce* (needles and twigs) and *P. heldreichii* (needles and twigs), identify the main components and compare the chemical composition of these *Pinus spp*. with previous studies.

## Materials and methods

### Chemicals and reagents

Dulbecco’s minimum essential medium (DMEM), foetal bovine serum (FBS), l-glutamine and a penicillin/streptomycin mixture were obtained from Life Technologies (Carlsbad, CA). Raw 264.7, HeLa, CaCo-2 and MCF-7 cell lines were purchased from American Type Culture Collection (ATCC-TIB-71, ATCC-CCL-2, ATCC-HTB-37, ATCC-HTB-22). Thiazolyl Blue Tetrazolium Bromide (MTT) was obtained from Sigma-Aldrich. Hydrochloric acid, disodium hydrogen phosphate, potassium chloride, sodium chloride and sodium dodecyl sulphate (SDS) were purchased from Merck (Darmstadt, Germany). Potassium dihydrogen phosphate was obtained from Sigma-Aldrich (St. Louis, MO).

### Plant material

Needles, twigs and cones of *P. mugo, P. peuce* and *P. heldreichii* collected from July to September 2013 from wild populations in Kosovo. The populations were originated from ‘Sharri’ National Park, Mountain (Mt.) Ošljak (*P. mugo* – 42°14′41E″N, 20°55′06″E, 1740 m a.s.l; *P. peuce* – 42°15′11″N, 20°54′56″E, 1627 m a.s.l.; *P. heldreichii* – 42°11′02″N, 20°54′56″E, 1555 m a.s.l). The collection sites were recorded using a Global Positioning System (GPS) receiver (GARMIN, eTrex^®^ 30). Two to four replicate samples of needles, twigs and cones were analyzed, each sample was gathered from 2–3 individual plants from each population. Samples were distilled and analyzed separately. Voucher specimens of each population were deposited at the Herbarium of the Department of Biology, University of Prishtina and authenticated by the University of Prishtina staff botanist Avni Hajdari.

### Essential oil extraction

The plant material was air-dried in shade at room temperature and cut into small pieces (<0.5 cm). Separated needles, twigs (only wooden parts) and cones were subjected to essential oil distillation. For distillation, 50 g of dry tissue was placed into 0.5 L of water in a 1 L flask and distilled in a Clevenger apparatus for 3 h at a rate of 3 mL/min. The samples were stored in the dark at −18 °C in the freezer pending further analysis. The yield of essential oil is expressed as a percentage of the mass of the essential oil with respect to the air-dried material (% v/w of dried material).

### Gas chromatography/flame ionization detector (GC/FID) and gas chromatography/mass spectrometry (GC/MS) analysis

GC/FID analyses were performed using an Agilent 7890 A GC System equipped with an FID detector (Agilent Technologies, Santa Clara, CA). The separation was conducted on an HP-5MS column 30 m × 0.25 mm with 0.25 μm film thickness. Helium was used as carrier gas with an initial flow rate of 0.6 mL/min and subsequently at a constant pressure of 50 psi. The front inlet was maintained at 250 °C in a split ratio of 50:1. The GC oven temperature was increased from 60 °C to 260 °C at a rate of 5 °C/min and the FID operated at 280 °C with an air flow of 350 mL/min and a hydrogen flow of 35 mL/min. The injection volume was 1 μL.

GC/MS analyses were performed using an Agilent 7890 A GC system coupled to a 5975 C MSD (Agilent Technologies). The ionization energy was 70 eV with a mass range of 40–400 *m*/*z*. The separation was conducted with the same column and temperature program as for the analytical GC.

Identification of the components of the essential oil was made by comparing their Kovats retention indexes with those in the literature (Adams 1997). The calculation of the Kovats index was made based on a linear interpolation of the retention time of the homologous series of *n*-alkanes (C9-C28) under the same operating conditions. Furthermore, the components were identified by comparing the mass spectra of each constituent with those stored in the NIST 08.L and WILEY MS 9th database and with mass spectra from the literature (Adams 1997). The percentage composition of the oils was computed by the normalization method from the GC peak areas, calculated as the mean of three samples, without correction factors.

### Assay of anti-inflammatory activity

To examine the anti-inflammatory effect of the EOs, an assay with murine macrophages was performed as described in Mueller et al. (2010). RAW 264.7 cells were seeded at a density of 2 × 10^6^ cells/mL into 24 well plates and incubated for 24 h at 37 °C, using DMEM (+10% FBS, 100 U/mL penicillin/streptomycin, 4 mM l-glutamine) as culture medium. On the following day, the oils were added in concentrations ranging from 0.0001–0.2% and the cells were further incubated for 3 h. The macrophages were then treated with LPS at a final concentration of 1 μg/mL to stimulate cytokine production. After 24 h of incubation, the supernatant was removed and centrifuged to remove any cell residues. The IL-6 secretion was quantified using an ELISA, according to the manufacturer’s protocol (eBioscience).

In parallel, the viability of the LPS-stimulated cells was assessed using an MTT assay, based on the reduction of MTT to formazan by living cells. 50 μL of MTT solution (5 mg/mL in 1 × phosphate-buffered saline, PBS) was added to the cells and the plate was incubated for 2 h at normal culture conditions. The supernatant was then removed and the cells were lysed with a buffer, containing 10% SDS in 0.01 N HCl. Using a microplate reader (Infinite M200, Tecan, Austria), the optical density was measured at 570 nm, corrected by a reference wavelength of 690 nm.

To reduce any variation from differences in cell density, the ELISA results were normalized to the MTT values. The concentration of cytokines of the positive control (cells only treated with LPS) was defined as 100%. All results from the tested oils were then calculated as a percentage of the positive control. The entire inflammation assay was performed in triplicate on independent days.

### Assay of cytotoxicity to cancer cell lines

To determine cell viability and thus potential cytotoxicity of substances to three different cancer cell lines (HeLa, CaCo-2, MCF-7), an MTT-assay was performed as described above, but in 96-well plates. Cells were seeded at a density of 2 × 10^6^ cells/mL and incubated for 24 h before addition of the test substances at concentrations ranging from 0.001–0.1% and further incubation of 24 h. Then, the wells were loaded with 10 μL MTT solution. After another 2 h of incubation, the supernatant of each well was removed and the cells were lysed with 100 μL of lysis buffer. The absorption was measured at 570 nm and corrected by a reference wavelength of 690 nm. The amount of cells of the positive control (cells only incubated with DMEM) was defined as 100%. The results from the test substances were calculated as a % of the positive control. The entire assay of cytotoxicity was performed in triplicate on independent days.

### Statistical calculations

The results of a triplicate are expressed as means ± standard deviation. IC_50_ was determined using a logistic dose-response model of the Table Curve 2 D software (Systat Software, San Jose, CA).

## Results

### Essential oil composition

The yield and chemical composition of oils from three *Pinus* spp. are presented in Table 1. In total, 58 compounds of the EOs were characterized from *P. mugo* needles, twigs and cones, representing 97.2, 96.1 and 72.1% of the total identified components, respectively. The main components detected in needles, twigs and cones oils are: δ-3-carene (19.3, 28.1 and 15.8%); α-pinene (21.3, 8.2 and 4.1%); (*E*)-caryophyllene (6.7, 4.1 and 20.2%); limonene (3.7, 19.5 and 2.2%); β-pinene (3.7, 11 and 1.6%), linalool-acetate (0.1, 7.2 and 1.2%), germacrene D (6.1, 0.4 and 1.2%), linalool (0.2, 0.1 and 4.6%).

**Table 1.** Composition [%] of the EOs of *P. mugo*, *P. peuce* and *P. heldreichii*.

		*P. mugo*			*P. peuce*		*P. heldreichii*	
Constituents^a^	KI^b^	Needles	Twigs	Cones	Needles	Twigs	Needles	Twigs
Tricyclene	926	0.80	0.17	tr	0.53	0.13	0.16	tr
α-Thujene	930	1.44	0.26	0.20	–	–	–	–
α-Pinene	940	21.34	8.20	4.10	36.79	15.96	10.57	14.32
Camphene	948	2.82	0.65	0.30	8.04	2.00	0.64	0.43
Thuja-2,4(10)-diene	960	0.11	0.54	0.70	–	–	–	–
Sabinene	975	0.95	1.12	0.40	0.06	0.35	tr	0.03
β-Pinene	979	3.66	10.99	1.60	13.00	21.48	1.66	0.89
Myrcene	990	1.94	2.36	1.10	0.79	1.98	1.56	1.86
α-Phellandrene	1005	0.26	0.38	tr	0.21	tr	tr	tr
δ-3-Carene	1011	19.29	28.05	15.80	0.20	0.86	0.11	tr
α-Terpinene	1018	0.37	0.23	0.20	tr	tr	–	–
*p*-Cymene	1024	0.32	0.38	0.30	–	–	–	–
β-Phellandrene	1029	–	–	–	6.07	35.82	–	–
Limonene	1031	3.67	19.50	2.20	–	–	43.93	64.22
β-*E*-Ocimene	1050	0.60	tr	tr	0.44	0.27	–	–
γ-Terpinene	1062	0.72	0.42	0.40	0.27	0.17	–	–
γ-Terpinolene	1088	3.60	2.62	1.10	0.29	0.35	0.20	0.05
Linalool	1098	0.24	0.12	4.60	0.69	0.21	–	–
α-Campholenal	1127	tr	0.31	0.20	tr	0.11	–	–
*E-*Pinocarveol	1139	0.16	0.20	0.20	0.44	0.24	0.43	0.04
Z-Verbenol	1141	–	–	–	0.32	0.15	–	–
Camphor	1146	0.42	0.29	0.30	–	–	–	–
Borneol	1165	0.60	0.57	1.00	0.11	0.22	0.16	tr
Terpinene-4-ol	1177	0.10	0.21	tr	tr	0.25	0.20	tr
*p*-Cymen-8-ol	1183	0.23	0.23	tr	–	–	–	–
α-Terpinol	1188	0.25	0.24	0.80	9.27	2.37	0.31	0.11
Methyl salicylate	1191	0.23	0.88	0.20	–	–	–	–
Myrtenol	1195	0.50	0.20	0.20	0.20	0.24	0.61	0.06
*E*-Piperitol	1208	–	–	–	1.20	0.29	–	–
Thymol methyl ether	1235	0.16	tr	1.10	–	–	–	–
Carvone	1243	–	–	–	–	–	0.22	0.14
Linalool acetate	1256	0.11	7.23	1.20	0.14	tr	–	–
Bornyl acetate	1285	3.03	tr	0.50	4.23	3.30	0.35	0.60
α-Copane	1376	0.14	0.19	0.20	0.23	0.36	0.16	tr
β-Bourbonene	1384	0.25	tr	tr	0.13	0.61	0.13	tr
β-Elemene	1391	1.46	0.86	0.30	tr	tr	0.18	tr
Longifolene	1407	0.33	tr	1.40	–	–	tr	1.31
Unknown 1	1420	tr	0.23	0.90	–	–	–	–
*E*-Caryophyllene	1418	6.68	4.05	20.20	tr	tr	4.40	0.33
β-Copaene	1432	0.33	0.23	0.50	tr	0.11	0.11	0.11
α-Humulene	1454	0.43	0.17	tr	tr	0.13	2.11	0.95
Aromadendrene	1458	1.07	0.88	3.20	–	–	–	–
*Z*-Muurola-4(14),5-diene	1466	0.37	0.05	0.20	0.23	1.01	tr	0.41
β-Chamigrene	1477	tr	0.27	0.30	–	–	–	–
γ-Muurolene	1479	0.87	tr	0.60	–	–	0.93	1.39
Germacrene D	1481	6.14	0.39	1.20	10.00	4.69	17.17	2.04
Bicyclogermacrene	1500	2.11	0.61	0.30	0.86	0.32	–	–
α-Muurolene	1500	0.71	0.20	0.30	–	–	0.22	0.14
γ-Cadinene	1519	0.90	0.19	0.30	0.29	0.81	0.40	0.19
β-Cadinene	1519	–	–	–	–	–	0.92	0.18
δ-Cadinene	1523	2.33	0.43	0.70	0.86	0.90	1.08	0.18
*trans*-Cadina-1,4-diene	1534	0.52	0.10	0.40	–	–	0.22	0.12
α-Cadinene	1538	0.41	0.31	0.70	0.31	tr	–	–
*E*-Nerolidol	1561	0.43	0.20	0.20	0.24	tr	–	–
Germacrene-4-ol	1582	tr	tr	tr	0.27	0.13	0.14	0.07
Spathulenol	1578	1.09	0.21	0.20	0.33	0.40	0.97	tr
Caryophyllene oxide	1583	1.49	0.29	0.40	0.14	0.22	3.11	2.09
Humolene epoxide II	1636	–	–	–	0.35	0.16	0.12	0.15
*Z*-Cadin-4-en-7ol	1636	–	–	–	0.51	0.42	0.65	0.07
*epi*-α-Cadinol	1640	0.99	tr	0.30	–	–	0.11	0.58
α-Muurolol	1645	0.18	0.14	1.30	0.19	0.00	0.29	0.09
α-Cadinol	1653	–	–	–	0.14	0.13	0.17	0.08
Khusinol	1680	–	–	–	–	–	0.20	0.67
Eudesma-4(15),7-diene-1-β-ol	1688	–	–	–	0.19	0.18	0.17	0.12
*E*-Nerolidylacetate	1717	–	–	–	–	–	tr	0.72
Oplopanone	1733	–	–	–	–	–	tr	0.65
Benzyl benzoate	1762	–	–	–	–	–	tr	0.44
Phenyl ethyl octanoate	1847	–	–	–	–	–	tr	0.59
3*Z*-Hexenyl cinnamate	1881	–	–	–	–	–	tr	0.43
Abietadiene	2087	tr	tr	0.20	–	–	–	–
Unknown 2	2116	tr	0.71	20.20	–	–	–	–
Unknown 3	2159	0.91	0.69	tr	–	–	–	–
Unknown 4		0.06	0.60	4.80	–	–	–	–
Total identified		97.15	96.12	72.1	98.55	97.32	95.09	96.83
Yield %v/w (min and max values)		0.5–0.6	1.1–1.2	0.4–0.5	0.7–1.0	3.0–3.3	0.2–0.3	0.8–1.2

a Compounds are listed in order of elution from an HP-5MS column and their percentages were obtained by FID peak-area normalization.

b Kovats indices calculated against a C9-C28 *n*-alkanes mixture on the HP5 MS column. tr: traces.

Forty-five compounds were characterized in the oils of needles and twigs of *P. peuce*, representing 98.6% and 97.3% of the total identified components, respectively. The major constituents identified on the needle and twig oils, respectively are: α-pinene (36.8% and 16%); β-phellandrene (6.1% and 35.8%); β-pinene (13% and 21.5%), germacrene D (10% and 4.7%); α-terpinol (9.3% and 2.4%); camphene (8% and 2%); bornyl acetate (4.2% and 3.3%).

The major constituents from 47 ones, identified from the EOs of *P. heldreichii* in needles and twigs, respectively are: limonene (43.9% and 64.2%); germacrene D (17.2% and 2%); α-pinene (10.6, and 14.3%); (*E*)-caryophyllene (4.4% and 0.3%); caryophyllene oxide (3.1% and 2.1%); α-humulene (2.1% and 1%). The constituents identified represent 95.1% and 96.8% of the total oils in needles and twigs.

### Anti-inflammatory effect

**Figure 1.**
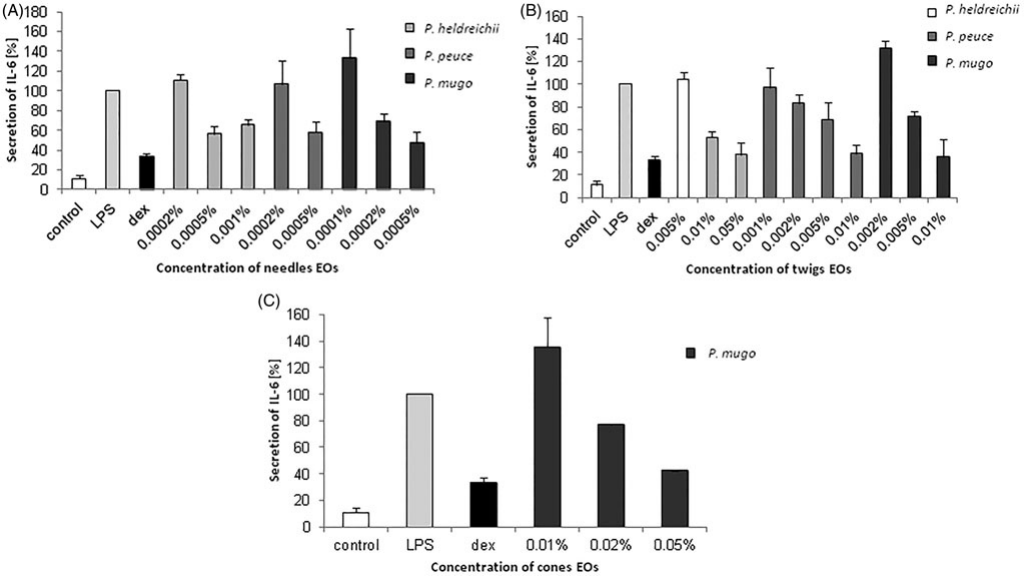
Concentration-depended reduction of IL-6 secretion of LPS-stimulated macrophages, as determined by ELISA, after treatment with (A) the needle and (B) twig oils of *P. heldreichii*, *P. peuce* and *P. mugo*, (C) the cone oils of *P. mugo* and 5 μM dexamethasone (dex).

EOs obtained from needles of *P. heldreichii*, *P. peuce* and *P. mugo* reduced the secretion of the pro-inflammatory cytokine IL-6 by at least 35% at a concentration of 0.001%, while the oils obtained from twigs of these three *Pinus* spp. and the cones of *P. mugo* decreased the secretion of IL-6 by at least 60% at a concentration of 0.01% for the oils obtained from twigs and 0.05% for the cones oils (Figure 1). IC_50_ values were determined with the needles EOs of *P. heldreichii* and *P. mugo* showing the lowest IC_50_ (0.0002), followed by *P. peuce* needles and twigs oils (>0.0005). EOs obtained from twigs of *P. heldreichii* and *P. mugo* showed an IC_50_ of >0.004 and the oils of cones of *P. mugo* had an IC_50_ of 0.02 (Table 2).

**Table 2.** IC_50_ values [%] of *P. heldreichii*, *P. peuce* and *P. mugo*.

	IC_50_ [%]			
		For cytotoxicity		
Substances	For IL-6 reduction	To HeLa	To CaCo-2	To MCF-7
*P. heldreichii* twigs	6 × 10^−3^	6 × 10^−2^	>1 × 10^−1^	>1 × 10^−1^
*P. heldreichii* needles	2 × 10^−4^	2 × 10^−2^	2 × 10^−2^	>1 × 10^−1^
*P. peuce* twigs	8 × 10^−4^	2 × 10^−2^	4 × 10^−2^	6 × 10^−2^
*P. peuce* needles	>5 × 10^−4^	7 × 10^−3^	2 × 10^−2^	6 × 10^−2^
*P. mugo* twigs	4 × 10^−3^	6 × 10^−2^	6 × 10^−2^	>1 × 10^−1^
*P. mugo* needles	2 × 10^−4^	3 × 10^−1^	2 × 10^−2^	3 × 10^−1^
*P. mugo* cones	2 × 10^−2^	>1 × 10^−1^	>1 × 10^−1^	>1 × 10^−1^

### Cytotoxicity to cancer cell lines

**Figure 2.**
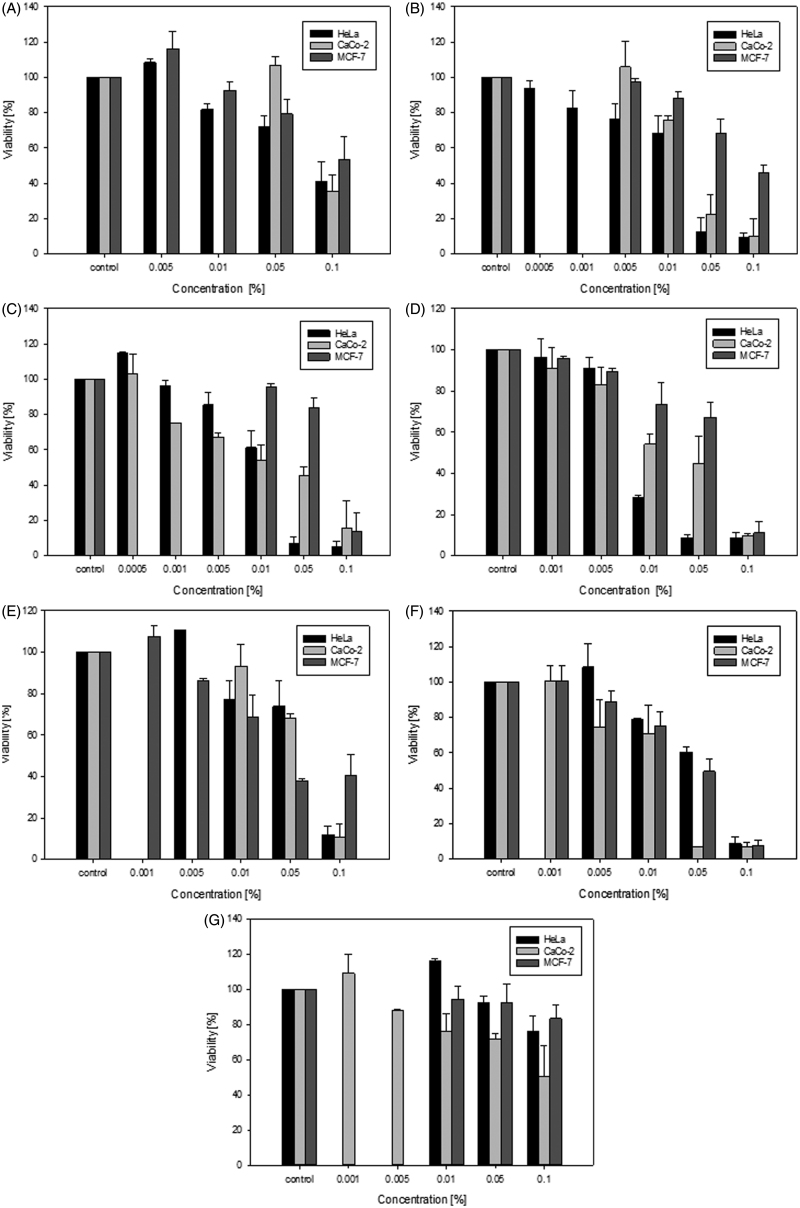
Concentration-depended cytotoxic effects of the oils of (A) *P. heldreichii* twigs, (B) *P. heldreichii* needles, (C) *P. peuce* twigs, (D) *P. peuce* needles, (E) *P. mugo* twigs, (F) *P. mugo* needles and (G) *P. mugo* cones on the three cancer cell lines HeLa, CaCo-2 and MCF-7, using an MTT-assay.

All EOs showed a significant cytotoxic effect on the cancer cell lines HeLa, CaCo-2 and MCF-7 (Figure 2). EOs obtained from *P. heldreichii* needles, *P. peuce* needles and twigs, and *P. mugo* needles and twigs at 0.1% concentration decreased the cell viability of HeLa by at least 90%, followed by oils obtained from *P. heldreichii* twigs with almost 60%. EOs of *P. mugo* cones had little cytotoxic effect with about 25%. IC_50_ values were determined with oils of the needles of *P. peuce* having the lowest value (0.007), followed by oils of the needles of *P. heldreichii* and oils of the twigs of *P. peuce* (0.02). Oils obtained from twigs of *P. heldreichii* and *P. mugo* showed an IC_50_ of 0.06, followed by *P. mugo* cones (>0.1) and *P. mugo* needles (0.3) (Table 2).

EOs obtained from *P. peuce* needles and from *P. mugo* needles and twigs led to a reduction of metabolic activity of CaCo-2 by 80–90%, followed by the oils of *P. heldreichii* twigs and oils of *P. mugo* cones with about 50–65%. IC_50_ values ranged from 0.02 for the oils of the needles of *P. heldreichii*, *P. peuce* and *P. mugo* to 0.04 for *P. peuce* twigs oils and 0.06 for *P. mugo* twigs oils. EOs of *P. heldreichii* twigs and *P. mugo* cones showed the highest IC_50_ with >0.1 (Table 2).

EOs of *P. peuce* twigs, *P. peuce* needles and *P. mugo* needles decreased cell viability of MCF-7 by at least 85%. EOs of *P. heldreichii* twigs, *P. heldreichii* needles and *P. mugo* twigs reduced metabolic activity of MCF-7 by 45–60%, while the EOs of *P. mugo* cones showed least cytotoxic effect to MCF-7 with only 15%. IC_50_ values ranged from 0.06 for *P. peuce* (needles and twigs) oils to >0.1 for *P. heldreichii* (needles and twigs) and *P. mugo* (twigs and cones) oils. Essential oil of *P. mugo* needles showed the highest IC_50_ with 0.3 (Table 2).

## Discussion

The EOs of the needles and twigs of *P. mugo*, *P. peuce* and *P. heldreichii* exert significant anti-inflammatory activity. In fact, the EOs from the twigs of all *Pinus spp.* showed a higher anti-inflammatory activity than their needles or cones.

α-Pinene, a major compound in all *Pinus spp.* may have an impact on the secretion profile of cytokines and thus, may play an important role in the anti-inflammatory activity. According to previous studies, the EOs of *Abies koreana*, which is rich in bornyl acetate, limonene and α-pinene, inhibited the IL-1β, IL-6 and TNF-α production in LPS-stimulated macrophages (Yoon et al. 2009). Furthermore, the EOs of *Olea europaea* L. (Oleaceae), with α-pinene as main constituent, exhibited significant anti-inflammatory activity, assessed by the inhibition of paw oedema (Haloui et al. 2010). Hippeli et al. (2004) hypotize, that the, up to now, unknown anti-inflammatory potential of *P. mugo* EOs and its main compounds α-pinene, limonene and δ-3-carene, is generated by the down-regulation of increased activity of neutrophils, thus reducing the inflammatory reaction in the body. They also assume that hydrogen peroxide, which was found in aged *P. mugo* oils, maintains the accumulation of microbicide agents. Further *in vitro* studies might confirm this hypothesis.

To date, the cytotoxic activity of the EOs from *P. heldreichii*, *P. mugo* and *P. peuce* on the cancer cell lines HeLa, MCF-7 and CaCo-2 has not been studied. EOs of *P. mugo* needles exerted the highest activity on the three cancer cell lines, while EOs of *P. mugo* cones exerted the least effect. According to the American National Center Institute, plant extracts that show cytotoxicity with an IC_50_ < 30 μg/mL can be considered as potential agents for the development of anticancer drugs (Suffness & Pezzuto 1990). Thus, on HeLa cell lines, EOs of *P. peuce* needles, *P. peuce* twigs and *P. heldreichii* needles with IC_50_ lower than 0.03% can be considered as promising agents for anticancer drug development while on CaCo-2 cell lines, the EOs from *P. mugo*, *P. peuce* and *P. heldreichii* needles can be considered as promising agents. The cytotoxicity of these EOs may be attributed to the major components, such as phellandrene, α-pinene, β-pinene, germacrene D and α-terpinol. These constituents are similar to the main compounds of *Myristica fragrans* Houtt. (Myristicaceae), which showed cytotoxic activities on CaCo-2 cell lines in a previous study (Piras et al. 2012). On MCF-7 cell lines, the highest activity was exerted by the EOs of *P. mugo* needles, followed by *P. peuce* needles and twigs. This effect can be attributed to the high amount of α-pinene in the oils, which showed anti-proliferative activities on MCF-7 cell lines in a previous study (Cole et al. 2007).

Even though the EOs obtained from twigs and needles of *P. heldreichii* were rich in limonene, with higher concentration in *P. heldreichii* twigs, the better results were achieved from *P. heldreichii* needles. Thus, limonene was not the main responsible compound for the activity towards HeLa, MCF-7 and Caco-2 cell lines. These results indicate that the higher activity of *P. heldreichii* needles are related to other main components such as germacrene D and β-pinene. Similar results were earlier reported, showing little cytotoxic activity of EOs rich in limonene (Bakarnga-Via et al. 2014). This is in contrary to other studies, showing good cytotoxic activity of limonene (Miller et al. 2013). These finding suggest that the cytotoxic activity of the EOs are not always related to the main components only, indicating that the activity is related to the synergism or cumulative effect of different EO components, including the components in trace (Bakkali et al. 2008).

Moreover, the chemical composition of the EOs from *P. mugo* needles, twigs and cones showed differences to each other. The major compounds of the essential oil in the needles are α-pinene and δ-3-carene (21.3% and 19.3%), which is similar to the *P. mugo* essential oil compositions reported by Hajdari et al. (2015) [α-pinene (17.0-24.5%), followed by δ-3-carene (15.5-27.9%), germacrene D (4.0-9.9%) and (*E*)-caryophyllene (4.3-9.0%)] and Stevanovic et al. (2005) [δ-3-carene (23.9%), *α*-pinene (17.9%)]. The major compounds on twigs EOs were δ-3-carene (28.1%), limonene (19.5%) and β-pinene (11.0%), similar to essential oil compositions reported by Hajdari et al. (2015) [3-δ-carene (24.0-51.7%) followed by limonene + β-phellandrene (12.7-24.3%), (*E*)-caryophyllene (4.0-10.9%), β-pinene (2.2-15.4%) and α-pinene (4.5-8.8%)], while in cone EOs the major compounds were (*E*)-caryophyllene (20.2%) and δ-3-carene (15.8%). Similar chemical composition was previously reported by Hajdari et al. (2015) [δ-3-carene (10.5-31.5%), followed by (*E*)-caryophyllene (10.4-27.0%)]. These essential oil compositions are very different to previous studies, which reported α-pinene (33% from *P. mugo* in Scotland) and bornyl acetate (11.5% from *P. mugo* in Italy) as main components, (Tsitsimpikou et al. 2001; Venditti et al. 2013). The different origins of the collected plants might explain these disagreements.

Limonene is the major compound in both, needle and twig EOs from *P. heldreichii*, with higher amount present in the twigs, followed by α-pinene and germacrene D, which is confirming to the results by Nikolić et al. (2007) who identified the same most abundant compounds in needle EOs in populations of Mts. Lovcen, Zeletin and Bjelasica; Montenegro, and Mt. Zlatibor to Mt. Pester; Serbia. There is a partial divergence to the dominant constituents (germacrene D > limonene > α-pinene) found by Nikolić et al. (2015) in needle EOs in populations of Mts. Ošljak and Galičica; Macedonia. These results are also similar to the investigation of EOs of needles, twigs and twigs with needles of *P. heldreichii* populations from Mt. Prenj (Bosnia and Herzigovina) by Chalchat et al. (1994) and to the research about the EOs of needles of *P. heldreichii* populations from two different locations in Serbia by Simić et al. (1996), which also show the highest amounts in limonene and germacrene D.

The dominant compounds in the EOs of the needles from *P. peuce* was found to be α-pinene, followed by β-pinene, germacrene D and α-terpinol, which is similar to the study of Nikolić et al. (2008) [α-pinene > germacrene D > camphene > β-pinene in populations of Mts. Zeletin and Sjekirica; Montenegro, and Mt. Mokra Gora; Serbia], Nikolić et al. (2014) [α-pinene > germacrene D > β-pinene > camphene in populations of Mts. Ošljak and Pelister; Macedonia], Karapandzova et al. 2010, 2011) [α-pinene > germacrene D > bornyl acetate > β-pinene in populations of Mt. Pelister; Macedonia; α-pinene > germacrene D > β-pinene > bornyl acetate in populations of Mts. Nidze and Shara; Macedonia; α-pinene > germacrene D > β-pinene > (*E*)-caryophyllene in populations of Mt. Karadzica; Macedonia] and Koukos et al. (2000) [α-pinene > β-pinene > citronellol in populations of Rhodope Mountains; northern Greece]. α-Pinene was identified as the main compound in the needles EOs of *P. peuce* collected from the Sar Planina mountain in Serbia, followed by γ-muurlene, δ-3-carene, sabinene, bornyl acetate and limonene (Simić et al. 1995). In fact, Simić et al. (1995) used a SE-30 capillary column, which could partially explain the disagreements in the main compounds (high amounts of γ-muurlene, δ-3-carene and sabinene) compared to the presented results and cited findings. In the twigs EOs of *P. peuce* the major compounds were found to be β-phellandrene followed by β-pinene and α-pinene which is similar to the study by Koukos et al. (2000) [β-phellandrene > citronellol > β-pinene > α-pinene in populations of Rhodope Mountains; northern Greece].

All these differences in the cytotoxic effects and anti-inflammatory activity are related to the factors influencing the chemical composition of the EOs, reported by Hussain et al. (2008) and Raut and Karuppayil (2014). Thus, these factors should be considered for characterization of the biological properties of plants harvested in different countries.

## Conclusions

The major compounds of the EOs in the needles were α-pinene and δ-3-carene, in the twigs the main compounds were δ-3-carene, limonene and β-pinene, while in cone EOs the major components were (*E*)-caryophyllene and δ-3-carene. The EOs of the twigs of all three *Pinus* spp. and the cones of *P. mugo* showed the most significant anti-inflammatory activity in LPS-stimulated macrophages. The EOs of the needles and twigs from *P. peuce* and *P. mugo,* as well as the needles of *P. heldreichii,* exerted the strongest cytotoxic activity on HeLa and CaCo-2 cell lines. On MCF-7 cell lines, the highest activity was shown by *P. peuce* (twigs and needles) and *P. mugo* needles. These EOs should be considered as potential agents for the development of new phytotherapeutic anticancer medicine.
